# Supplementary material for: The Construction and Exploration of a Comprehensive MicroRNA Centered Regulatory Network in Foxtail Millet (Setaria italica L.)
Source: Front Plant Sci. 2022 May 6;13:848474. doi: 10.3389/fpls.2022.848474 (PMC9121102; doi:10.3389/fpls.2022.848474)
Supplement: Supplementary file 2 [file Data_Sheet_2.zip › Sit-miRN2213_Seita.1G034300.1_1353_TPlot.pdf]

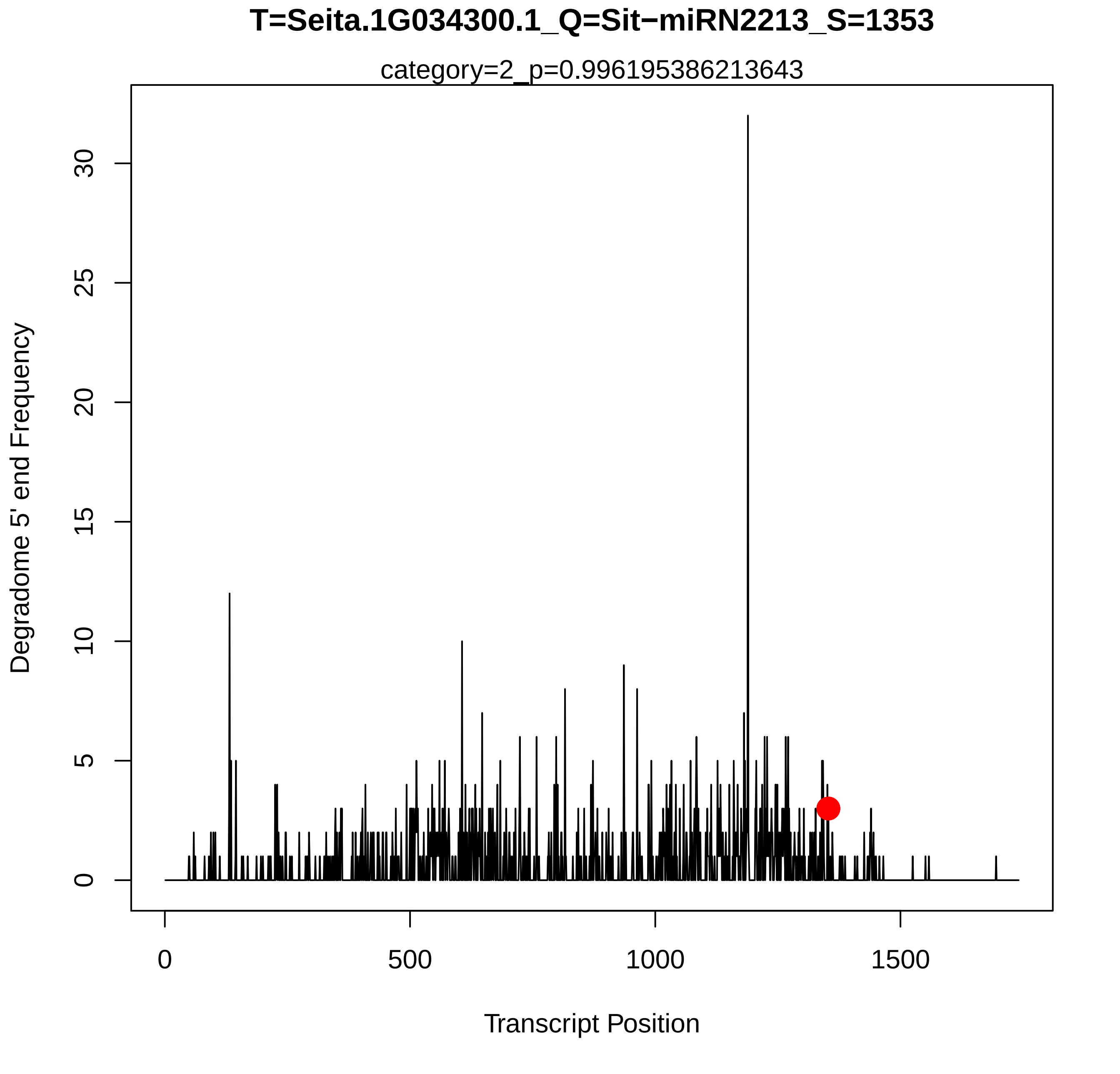
T=Seita.1G034300.1_Q=Sit−miRN2213_S=1353
category=2_p=0.996195386213643
Degradome 5' end Frequency
0
5
10
15
20
25
30
0
500
1000
1500
Transcript Position
